# Relationships between physical fitness and match running demands during a futsal congested-weeks training camp

**DOI:** 10.5114/biolsport.2024.134760

**Published:** 2024-03-06

**Authors:** Francisco Tomás González-Fernández, Halil Ibrahim Ceylan, Rui Miguel Silva, Filipe Manuel Clemente, Pedro Bezerra, Yung-Sheng Chen

**Affiliations:** 1 Department of Physical Education and Sports, Faculty of Sport Sciences. University of Granada, Granada 18071, Spain; 2 Physical Education and Sports Teaching Department, Kazim Karabekir Faculty of Education, Ataturk University, Erzurum, 25240, Turkey; 3 Escola Superior Desporto e Lazer, Instituto Politécnico de Viana do Castelo, Rua Escola Industrial e Comercial de Nun’Álvares, 4900-347 Viana do Castelo, Portugal; 4 Sport Physical Activity and Health Research & Innovation Center, Viana do Castelo, Portugal; 5 Gdansk University of Physical Education and Sport, 80-336 Gdańsk, Poland; 6 Department of Exercise and Health Sciences, University of Taipei, Taipei 111, Taiwan; 7 Exercise and Health Promotion Association, New Taipei City 241, Taiwan; 8 Tanyu Research Laboratory, Taipei 112, Taiwan

**Keywords:** Match load, Exercise intensity, Locomotion profile, Body composition, Indoor sports

## Abstract

This study examines the relationships between body composition, anthropometry and physical fitness measures, and internal and external load (match physical and running demands) during a congested period of an overseas futsal training camp. Eleven under-20 national futsal players participated in a eleven-day training camp. During the matches, exercise heart rate (HR) and locomotion profiles were recorded via a Polar Team Pro system as the players’ internal and external load. The friendly matches were scheduled on the training camp’s 2nd, 4th, 6th, 8th, and 10th days. A repeated-measures analysis of variance (ANOVA) and Pearson’s correlation coefficient were used for statistical analysis. The results revealed significant differences in sprint (*F* = 2.74, *p* = 0.04, η^2^ = 0.21), accelerations (*F* = 3.63, *p* = 0.04, η^2^ = 0.27), and decelerations (*F* = 2.73, *p* = 0.04, η^2^ = 0.21) performance between the five matches (*p* < 0.05). Also, the overall body composition and physical fitness measures had large to very large correlations with match running demands during congested periods of futsal competition (*p* < 0.05). In conclusion, the baseline body composition and the overall physical fitness measures may be essential factors in match running demands during a congested futsal match period. Futsal players who perform better in the 30–15 Intermittent Fitness Test can sustain a greater volume of high-intensity accelerations during a congested period.

## INTRODUCTION

Futsal is a dynamic, intermittent, fast-paced team sport played indoors with five players on each side (5-a-side), imposing high physical, technical, and tactical demands [1–3]. The smaller dimensions of the playing field and the option of unlimited player substitutions in futsal cause players to exhibit high-level physical performances during a match, including high oxygen uptake (VO_2_), short explosive movements (sprints), repeated plyometric requirements, accelerations, rapid changes in direction, and other high-intensity activities during transition phases [1, 4–8].

During a significant portion of a futsal game (more than 80%), players exceed 85 or 93% of their maximum heart rate (HR_max_: ~193 ± 8.39 bpm) [1, 9–11], with a corresponding blood lactate concentration ranging from 4.2 to 21.8 mmol/L [9, 10]. Further, time-motion analysis showed that professional futsal players covered a mean distance of 4000 m (117.3 m per minute), with about 28.5% of this distance being medium-intensity activity, 13.7% (1232 m), high-intensity activity (speed ≥ 15 km · h^−1^, 571 m), and 8.9% sprinting (speed ≥ 25 km · h^−1^, 349 m) during the games [1]. Another study noted that elite futsal players covered an average of 121 metres per minute, and sprints and high-intensity runs made up 5% and 12% of the total playing time, respectively [10]. Moreover, a futsal player performs sprints or repetitive high-intensity activities that heavily strain the aerobic and anaerobic pathways every 79 seconds for more than 80% of the time spent on the field [1, 6, 10].

Regarding physical fitness, maximal VO_2_ (V̇O_2max_) or aerobic capacity is considered a distinguishing factor in sustaining the physical demands and match performance, depending on the level of competition, as stated above [12]. Enhanced aerobic capacity helps with faster recovery between efforts and even after fatigue, delays the onset of fatigue in the second half of the match, improves lactate removal and phosphocreatine regeneration, increases the potential to cover longer distances in high-intensity runs and sprints, and contributes to being more involved in the game [7, 13, 14].

Body composition and anthropometric variables (height, body mass, lean body mass, and fat mass) play a fundamental role in maintaining high-level physical demands and match the above activity profile. Body fat percentage can limit an athlete’s ability to perform explosive movements in team sports [15]. Typically, elite male futsal players display low body fat around 10–11% [16, 17], 12% [18], and 15% [19]. Previous studies have found that high-fat mass had a negative effect on match performance, especially on sprint distance [20, 21], high-speed running distance [22], change of direction or agility [20, 21], and aerobic capacity [23, 24] in soccer players. Moreover, recent studies indicated a positive correlation between the anthropometric parameters with a higher percentage of muscle mass and V̇O_2max_ [20, 21], and strength production characteristics of athletes [25].

Similar to soccer, international futsal tournaments and playoffs for major futsal leagues worldwide are played during congested schedules, with very little time for players to rest and recover between matches. Indeed, exposure to two or three matches a week can increase the burden and stress on players, leading to residual fatigue and an increased risk of injury [26–28]. Furthermore, various performance outputs and muscle functions can be affected due to the elevation of markers associated with inflammation and muscle damage [29, 30]. Previous studies have found that congested schedules decreased muscle contractile function or muscle stiffness [30, 31] and hamstring strength performance [32] due to cumulative fatigue and lack of muscle recovery. Thus, there is a need to understand better how players participating in multiple matches within a week cope with the external demands placed upon them [33].

The matches played during congested weeks significantly impact external load parameters in professional soccer players [29, 33, 34]. However, there is a need for more research on the effects of playing matches during a congested period in futsal, and the uncertainty regarding its impact remains. Despite the limited number of studies that have examined the match demands of professional male [1, 7, 10, 28, 35, 36] and female futsal players [37], these studies could contribute not only to a better understanding of the workload and intensity requirements of players during the game but also to the improvement of their performance, and to minimize the risk of non-functional overreaching, overtraining, and injury [38]. For instance, a study showed that the match intensity of Oceania futsal players, evaluated through their HR, recovery kinetics, and health status, remained unchanged during a 4-day FIFA futsal tournament. However, later in the study, it was observed that despite a slight increase in players’ subjective perception of effort between matches, there was a decrease in sprint activity [11].

It was previously suggested that futsal players with high training load and aerobic and anaerobic capacity and power tend to perform at high intensities during a 4-day FIFA futsal tournament and take longer to recover from these intense efforts (intensity and recovery kinetics via HR measurements) [11]. In a study conducted on professional soccer players, a strong positive correlation was observed between the maximum aerobic speed (MAS) achieved during the final stage of the 30–15 Intermittent Fitness Test (IFT) and external load measures such as total distance, high-intensity running, accelerations, and decelerations [39]. In a recent study, professional soccer players with lower fat content and higher aerobic capacity covered longer distances in sprints and high-speed running (HSR) during official matches [22]. Lastly, the available evidence from a previous study suggested that the 30-m sprint test might be predicted by the session rate of perceived exertion (RPE), average and HR_max_, total distance covered per minute, maximal speed, and average speed in young national futsal players [18].

Considering the studies mentioned above, there is a need for more research examining the link between body composition, aerobic capacity, and external load metrics during futsal matches played in congested weeks. Therefore, closely monitoring external load during matches in very short and congested fixtures can provide valuable information to coaches and sports scientists to optimize players’ recovery and performance and design appropriate training programmes.

This study has two objectives: (1) to analyse differences in match physical and running demands among matches (from match 1 (M1) to match 5 (M5)); and (2) to examine the relationships between body composition, anthropometry, and physical fitness measures, and match physical and running demands during a congested week of an overseas futsal training camp. We assumed there would be variations in external load parameters during five matches played in a congested period (11 days). Additionally, physical fitness and body composition would positively affect the external load parameters during the congested period.

## MATERIALS AND METHODS

### Experimental Approach to the Problem

**FIG. 1:**
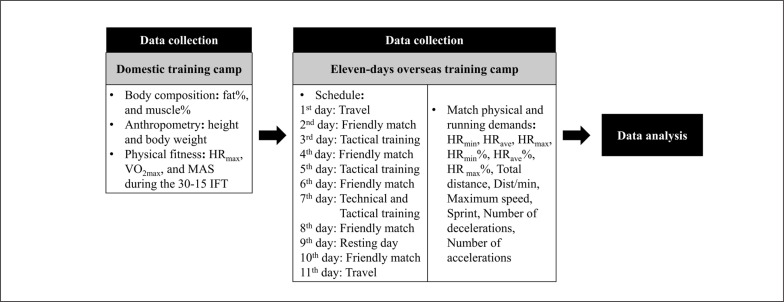
Flowchart of experimental design of the study.

This is an observational study during an eleven-day futsal training camp in Luso, Portugal, two weeks before the Asian under-20 Futsal Championship final. The training camp consisted of 5 friendly match days played with the regional futsal clubs, two tactical training days (3^rd^ day, 82 min and 5^th^ day, 72 min), 1 technical and tactical training day (7^th^ day, 80 min), and 1 resting day (9^th^ day of the camp). The friendly matches were scheduled on the training camp’s 2^nd^ (Atlético Luso U-20, 4:2. win), 4^th^ (ACR Vale de Cambra U-20, 13:5. win), 6^th^ (CS São João U-20, 1:2. lost), 8^th^ (União de Chelo U-20, 1:2. lost), and 10^th^ (Burinhosa U-20, 6:1. win) day. The exercise HR and locomotion profile were recorded during the matches as the players’ internal and external loads, respectively. Data extracted from three goalkeepers were excluded from the statistical analysis. Figure 1 presents the flowchart of the experimental design of the study.

### Participants

Eleven outfield players in the Chinese Taipei under-20 national futsal team voluntarily participated in this study (See Table 1). The inclusion criteria were based on: (i) being present at all sessions; (ii) not being injured in the two weeks preceding the training camp. (iii) not being injured or ill during the two weeks of observation; (iv) players who were absent for more than two training sessions were excluded from the sample. The players signed informed consent forms and were familiar with the testing protocol. The study was approved by the institutional board of the Human Ethics Committee at the University of Taipei (UT-IRB-2018-068) and abided by the Declaration of Helsinki and its later amendments in 2013.

**TABLE 1:** Anthropometrical measures and physical fitness of the participants.

	Mean ± SD	Min	Max
Age (yrs)	18.6 ± 0.7	18.0	20.0
Height (cm)	170.8 ± 7.6	157.0	181.0
Body weight (kg)	64.1 ± 8.4	51.0	75.9
Fat (%)	12.7 ± 3.0	9.3	20.5
Muscle (%)	43.2 ± 2.4	37.9	46.3
HR_max_ (bpm)	196.2 ± 8.8	173.0	203.0
V̇O_2max_ (ml/kg/min)	51.80 ± 3.8	45.8	56.6
30–15 IFT (km/h)	19.3 ± 1.6	17.0	21.0

Note: SD: Standard deviation; Min: Minimum; Max: Maximum; yrs: Years; cm: Centimeters; K: Kilograms; HR_max_; Heart rate maximum; V̇O_2max_: Maximal oxygen uptake. 30–15 Intermittent Fitness Test: 30–15 IFT.

### Procedure

Before international travel, the players’ height, body weight, and body composition were determined using a stadiometer (Seca 213, SECA, Germany), an electrical weight scale (Xyfwt382, TECO, Taiwan), and a skinfold caliper (Lange Skinfold Caliper, BETA Technology, USA), respectively. A team sports trainer used four skinfold thicknesses to determine the percentage of body fat and muscle. The formula for calculating body composition was reported in our previous study [40]. Subsequently, the players performed the 30–15 IFT for physical fitness measures (HR_max_, V̇O_2max_, and MAS) in an indoor sports hall. A 10-min dynamic warm-up exercise was performed prior to the test. A portable telemetric HR monitor system (Polar team Pro, Polar Electro, Kempele, Finland) was used to determine the HR_max_ during the 30–15 IFT. Our recent publication also reported the testing procedures to determine the players’ physical fitness [18].

On match days, the Polar Team Pro HR monitor system was used to record the exercise HR (HR_min_, HR_ave_, HR_max_, HR_min_%, HR_ave_%, and HR _max_%). Before the match, all players were issued a Polar Team Pro sensor and HR strap for the entire futsal match. The players familiarized themselves with securing the sensor at the chest level. Furthermore, the locomotor demands were monitored using a Polar telemetric global positioning system (GPS) (Polar team Pro GPS, Polar Electro, Kempele, Finland) with a sampling frequency of 10 Hz. The Polar GPS was accurate and reliable in collecting measures of total distance (TD) and distances covered at different speed thresholds [41]. The following measures were extracted for each match: 1) TD; 2) dist/min (average distance covered per minute, Dist); 3) maximum speed (peak speed registered in the session, MS); 4) average speed (km/h^−1^, AS); 5) sprint (number of sprints > 25 km/h, S); 6) number of decelerations (-50.00 – -3.00 m/s², ND1); 7) number of decelerations (-2.99 – -2.00 m/s², ND2); 8) number of deceleration (-1.99 – -1.00 m/s², ND3); 9) number of decelerations (-0.99 – -0.50 m/s², ND4); 10) number of accelerations (0.50–0.99 m/s², NA1); 11) number of accelerations (1.00–1.99 m/s², NA2); 12) number of accelerations (2.00–2.99 m/s², NA3); 13) number of accelerations (3.00–50.00 m/s², NA4).

All sensors were synced to a Polar Team Pro dock after the matches. All data were uploaded to a secure cloud server and then exported to a laptop for data analysis. Data were excluded from statistical analysis when the detection of the HR response and locomotion record was lost (due to physical contact or vigour of physical activity).

### Statistical Analyses

The statistical analysis was conducted using Statistica software (version 13.1; StatSoft, Inc., Tulsa, OK, USA) and the tool Real Statistica Using Excel. The significance level was set at *p* < 0.001. Tests for normal distribution and homogeneity (Kolmogorov-Smirnov and Levene’s, respectively) were conducted on all metrics, and the data were found to have a normal distribution. Descriptive statistics were calculated for each anthropometric measure (age, height, body weight, fat%, and muscle%), physical fitness values during the 30–15 IFT (HR_max_, V̇O_2max_, and MAS), and match physical and running demands (N, D, HR_min_, HR_ave_, HR_max_, HR_min_%, HR_ave_%, HR _max_%, TD, Dist, MS, AS, S, ND1, ND2, ND3, ND4, NA1, NA2, NA3, and NA4) during the congested schedule of five futsal matches (matches 1–5: M1–M5). A within-subjects comparison was performed for the statistical approach. A repeated-measures analysis of variance (ANOVA) was used to analyse the differences in match physical and running demands during the congested schedule from M1 to M5. The effect size was determined with partial eta squared for Fs. Pearson’s correlation coefficient (*r*) was then used to examine the relationship between anthropometric measures and physical fitness values and match physical and running demands during the congested schedule of futsal matches. To interpret the magnitude of these correlations, the following criteria were used: *r* ≤ 0.1, trivial; 0.1 < *r* ≤ 0.3, small; 0.3 < *r* ≤ 0.5, moderate; 0.5 < *r* ≤ 0.7, large; 0.7 < *r* ≤ 0.9, very large; and *r* > 0.9, almost perfect [42]. Repeated measures correlation (rmcorr) was performed to examine the overall relationship or common intra-individual association between measures to adjust for inter-individual variability statistically. The 95% confidence interval (CI) was calculated for each rmcorr. The statistical analysis was conducted using Python software (version; 3.11.4) for rmcorr.

## RESULTS

Descriptive statistics were calculated for each variable to match physical and running demands (Table 2).

**TABLE 2:** Match physical and running demands profiles during congestive schedule.

	Match 1			Match 2			Match 3			Match 4			Match 5		
	Mean ± SD	Min	Max	Mean ± SD	Min	Max	Mean ± SD	Min	Max	Mean ± SD	Min	Max	Mean ± SD	Min	Max
**Substitution (n)**	5.3 ± 0.9	4.0	7.0	4.5 ± 1.0	2.0	6.0	5.7 ± 1.6	3.0	8.0	5.6 ± 2.1	2.0	8.0	5.6 ± 1.4	4.0	4.0
**Duration (min)**	273.0 ± 27.1	216.7	311.8	269.4 ± 47.5	216.0	389.6	246.5 ± 53.6	172.3	333.6	269.9 ± 38.5	193.0	321.6	235.3 ± 53.5	103.0	294.4
**HR_min._ (bpm)**	123.5 ± 10.5	106.0	136.0	123.9 ± 14.7	97.0	142.0	123.3 ± 13.7	101.0	148.3	121.4 ± 14.8	96.7	140.3	127.8 ± 10.1	106.5	141.0
**HR_ave_ (bpm)**	174.3 ± 7.3	161.0	183.3	174.0 ± 7.1	160.8	181.2	173.0 ± 6.9	160.3	181.6	173.8 ± 9.3	158.0	186.0	172.9 ± 7.2	157.5	187.5
**HR_max_ (bpm)**	190.4 ± 6.7	176.4	200.2	190.5 ± 6.0	176.0	199.2	190.2 ± 5.4	180.8	200.2	190.9 ± 8.1	175.0	200.6	187.2 ± 6.6	171.5	199.3
**HR_min_%**	63.5 ± 5.9	52.2	71.8	63.7 ± 8.0	47.8	76.0	63.5 ± 7.8	49.5	79.5	62.5 ± 8.2	47.7	75.0	65.5 ± 4.7	59.0	75.0
**HR_ave_%**	89.6 ± 4.7	81.6	97.3	89.5 ± 4.5	79.6	95.8	88.9 ± 4.8	78.8	95.5	89.4 ± 5.6	77.7	96.2	88.9 ± 3.7	85.3	97.3
**HR_max_%**	97.9 ± 3.6	91.8	103.3	97.9 ± 3.5	93.0	103.5	97.8 ± 4.5	91.8	104.6	98.2 ± 4.7	88.7	103.2	96.3 ± 4.0	92.0	103.8
**TD (m)**	499.1 ± 84.7	348.7	616.2	480.3 ± 112.9	357.5	759.4	453.0 ± 108.6	297.8	581.4	489.6 ± 98.1	354.8	634.0	425.2 ± 129.7	205.0	672.8
**Dist (m/min)**	108.8 ± 11.8	89.4	127.0	107.1 ± 10.1	91.5	123.8	110.4 ± 11.4	91.2	132.8	109.3 ± 14.3	89.0	138.2	108.2 ± 14.7	91.0	141.3
**MS (m/s)**	24.9 ± 1.7	22.0	27.2	24.8 ± 2.1	19.3	27.8	24.3 ± 1.7	21.7	26.6	25.2 ± 1.8	20.3	27.3	24.3 ± 2.6	18.2	28.0
**AS (m/s)**	6.6 ± 0.7	5.4	7.6	6.5 ± 0.6	5.5	7.4	6.6 ± 0.7	5.5	8.0	6.6 ± 0.9	5.4	8.30	6.5 ± 0.9	5.5	8.5
**S (m/min)**	1.3 ± 0.6	0.3	2.5	1.3 ± 0.8	0.0	2.8	1.4 ± 0.9	0.2	2.8	2.0 ± 1.0	0.0	3.6	1.5 ± 1.6	0.0	5.5
**ND1 (n)**	4.4 ± 1.7	2.2	8.3	4.2 ± 1.4	2.3	6.8	3.6 ± 1.6	2.0	6.2	4.6 ± 1.6	1.8	7.0	3.8 ± 2.1	2.0	9.5
**ND2 (n)**	11.4 ± 1.9	8.3	13.8	11.4 ± 2.7	7.6	16.4	11.2 ± 3.3	7.5	18.8	11.5 ± 2.7	7.0	17.3	10.2 ± 2.3	7.0	13.8
**ND3 (n)**	34.1 ± 4.9	26.8	41.8	33.0 ± 7	22.8	44.0	29.2 ± 6.7	22.8	43.0	32.5 ± 5.2	22.0	40.6	28.3 ± 7.1	15.0	42.6
**ND4 (n)**	22.0 ± 3.8	17.7	29.8	21.1 ± 6.3	13.8	37.4	19.1 ± 4.5	10.8	27.4	20.7 ± 5.1	13.0	29.8	17.9 ± 6.7	7.0	27.0
**NA1 (n)**	19.6 ± 3.7	13.0	25.4	19.1 ± 3.6	13.8	26.8	17.2 ± 5.1	11.0	24.6	18.2 ± 3.8	12.0	23.8	16.1 ± 3.8	9.0	23.2
**NA2 (n)**	31.1 ± 3.7	24.6	36.2	30.5 ± 5.2	25.0	39.8	27.3 ± 4.7	20.0	34.8	29.9 ± 4.5	19.0	33.8	25.6 ± 7.3	14.0	36.6
**NA3 (n)**	16.1 ± 2.56	11.0	20.0	15.7 ± 3.6	11.3	21.4	14.3 ± 4.2	9.2	23.0	16.5 ± 2.8	11.8	20.8	13.9 ± 3.5	9.0	21.5
**NA4 (n)**	0.1 ± 0.1	0.0	0.3	0.3 ± 0.3	0.0	1.0	0.2 ± 0.2	0.0	0.6	0.2 ± 0.3	0.0	1.0	0.2 ± 0.4	0.0	1.3

Note: SD: Standard deviation; Min: Minimal; Max: Maximal; N: Number of substitution; D: Duration; HR_min_: Heart rate minimum; HR_ave_: Heart rate average; HR_max_: Heart rate maximum; HR_min_%: Percentage of heart rate minimum; HR_ave_%: Percentage of heart rate average; HR_max_%: Percentage of heart rate maximum; TD: Total distance; Dist: Distance; MS: Maximum speed; AS: Average speed; S: Sprint; ND1: Number of decelerations (-50.00 – -3.00 m/s²); ND2: Number of decelerations (-2.99 – -2.00 m/s²); ND3: Number of decelerations (-1.99 – -1.00 m/s²); ND4: Number of decelerations (-0.99 – -0.50 m/s²); NA1: Number of accelerations (0.50–0.99 m/s²); NA2: Number of accelerations (1.00–1.99 m/s²); NA3: Number of accelerations (2.00–2.99 m/s²) and NA4: Number of accelerations (3.00–50.00 m/s²)

For comparative analyses, a repeated-measures ANOVA with mean match physical and running demands data during the congested schedule from M1 to M5 did not reveal significant main effects of match on N, D, HR_min_, HR_ave_, HR_max_, HR_min_%, HR_ave_%, HR_max_%, TD, Dist, MS, AS, S, ND1, ND2, ND4, NA1, NA3, and NA4 (*p* > .05). Crucially, the analysis revealed a significant main effect on S (*F =* 2.74, *p* = 0.04, η^2^ = 0.21), ND3 (*F =* 2.73, *p* = 0.04, η^2^ = 0.21), and NA2 (*F =* 3.63, *p* = 0.04, η^2^ = 0.27).

**FIG. 2:**
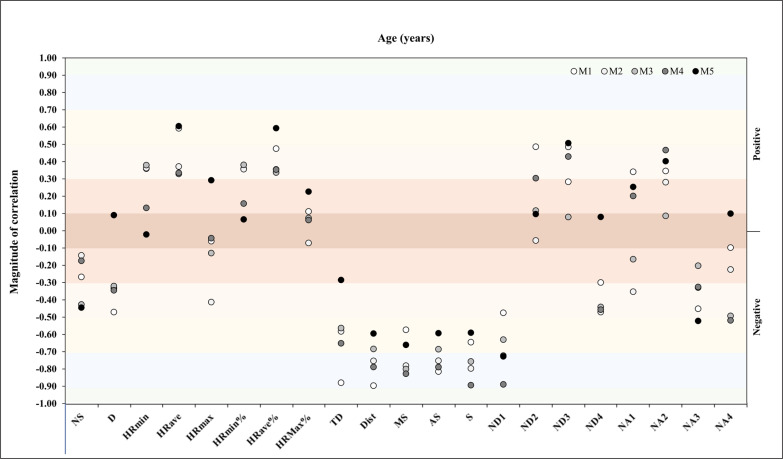
Correlation analysis between age and match physical and running demands.

A correlation analysis between age and match running demands during the congested schedule of futsal matches revealed in M1 a very large negative correlation with TD, Dist, and AS (*r* = -.88, *p* = .004, *r* = -.89, *p* = .003, *r* = -.81, *p* = .01, respectively). In M2, the analysis showed a very large negative correlation with Dist, MS, AS, S, and ND1 (*r* = -.75, *p* = .03, *r* = -.78, *p* = .02, *r* = -.75, *p* = .03, *r* = -.79, *p* = .02, *r* = -.72, *p* = .04, respectively). In M3, the analysis revealed a very large negative correlation with MS (*r* = -.80, *p* = .02) and S (*r* = -.75. *p* = .03) and in M4, the analysis revealed a very large negative correlation with Dist, MS, AS, S, and ND1 (*r* = -.78, *p* = .02, *r* = -.82, *p* = .01, *r* = -.79, *p* = .02, *r* = -.89, *p* = .003, *r* = -.89, *p* = .003, respectively) (Figure 2).

**FIG. 3:**
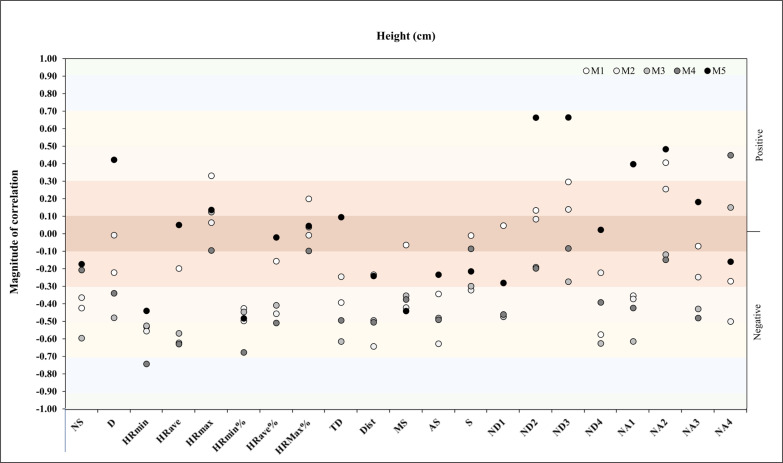
Correlation analysis between height and match physical and running demands.

A new correlation analysis was performed between height and match physical and running demands during the congested schedule of futsal matches and only showed in M4 a very large negative correlation with HR_min_ (*r* = -.74, *p* = .035) (Figure 3).

**FIG. 4:**
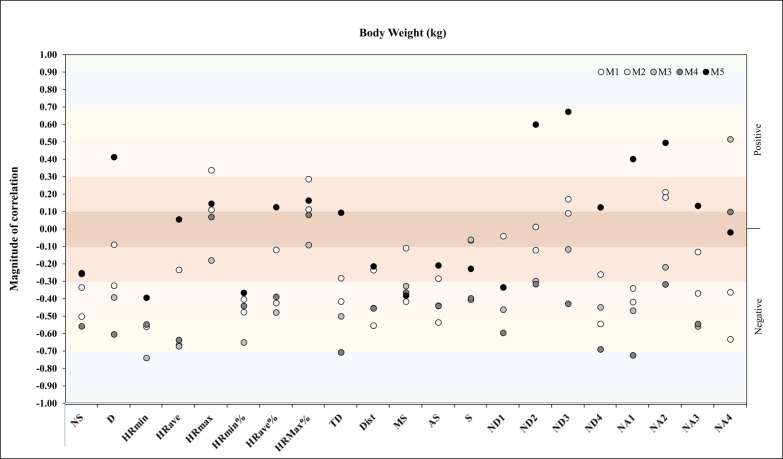
Correlation analysis between body weight and match physical and running demands.

Another correlation analysis was performed between body weight and match physical and running demands during the congested schedule of futsal matches. It revealed in M3 a very large negative correlation with TD (*r* = -.71, *p* = .05) and in M4 another very large negative correlation with HR_min_ (*r* = -.74. *p* = .04) (Figure 4).

**FIG. 5:**
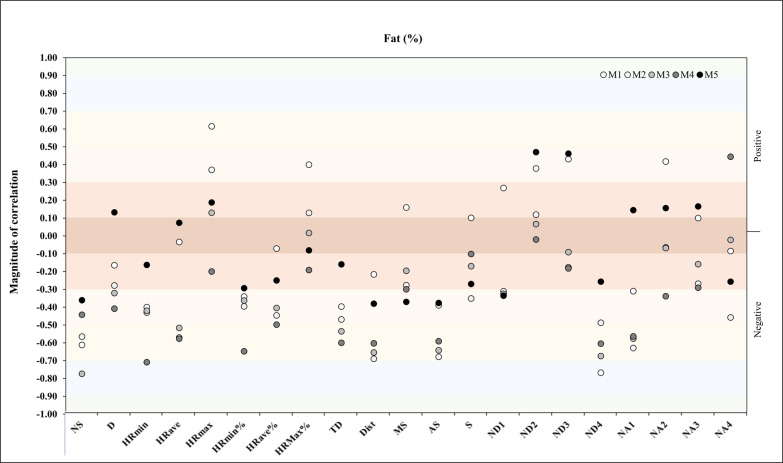
Correlation analysis between fat% and match physical and running demands.

A further correlation analysis was performed between fat% and match physical and running demands during the congested schedule of futsal matches. In M1, correlation analysis showed a very large negative correlation with ND4 (*r* = -.77, *p* = .03). In M3, the dataset revealed another very large negative correlation with N (*r* = -.77. *p* = .02), and in M4, the analysis showed a very large negative correlation with HR_min_ (*r* = -.71. *p* = .05) (Figure 5).

**FIG. 6:**
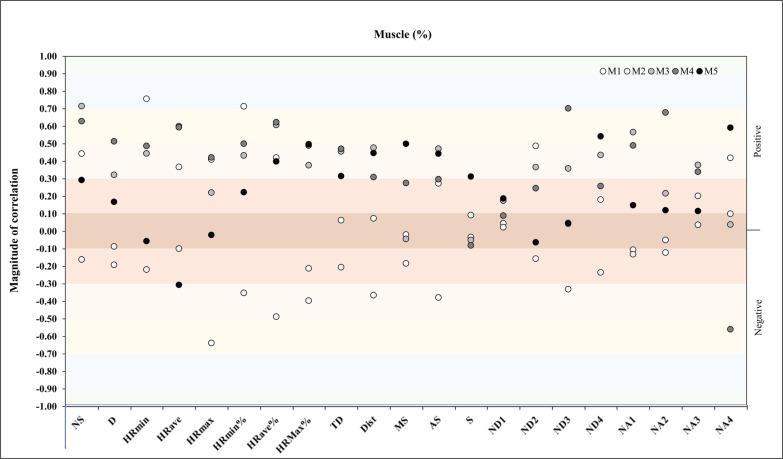
Correlation analysis between muscle% and match physical and running demands.

Another correlation analysis was performed between muscle% and match physical and running demands during the congested schedule of futsal matches. It revealed in M1 a very large positive correlation with HR_min_ (*r* = .76, *p* = .03) and HR_min_% (*r* = .71, *p* = .05), and in M3 a very large positive correlation with HR_min_ (*r* = .72, *p* = .03) (Figure 6).

**FIG. 7:**
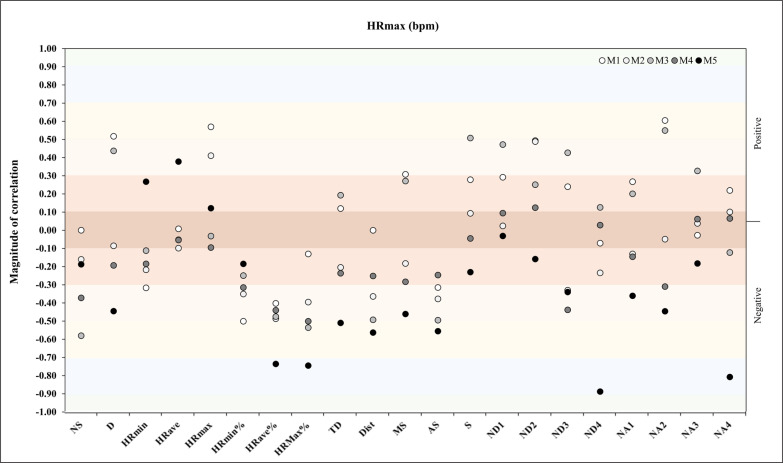
Correlation analysis between HR_max_ and match physical and running demands.

**FIG. 8:**
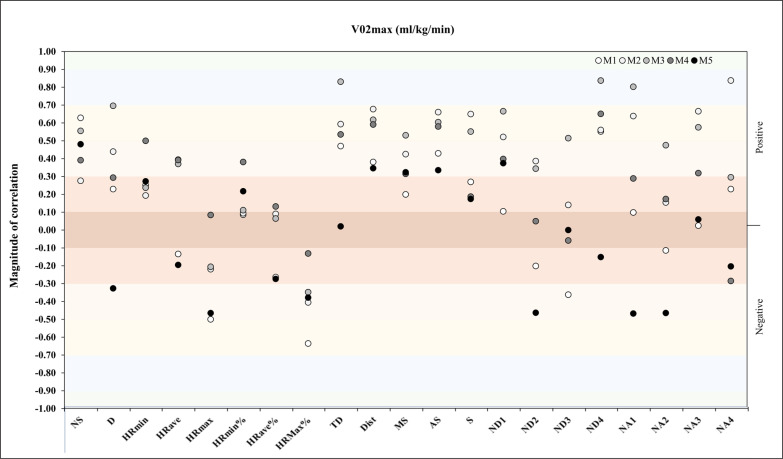
Correlation analysis between V̇O_2max_ and match physical and running demands.

Regarding HR_max_, in match 5, we found a very large negative correlation with ND4 (*r* = -.89, *p* = .01) and NA4 (*r* = -.81, *p* = .03) (Figure 7). In reference to V̇O_2max_, the analysis showed a very large positive correlation with NA4 (*r* = -.84, *p* = .01) and a very large positive correlation with TD, ND4, and NA1 (*r* = -.83, *p* = .01, *r* = -.84, *p* = .01, *r* = -.80, *p* = .02) in M2 and M3, respectively (Figure 8).

**FIG. 9:**
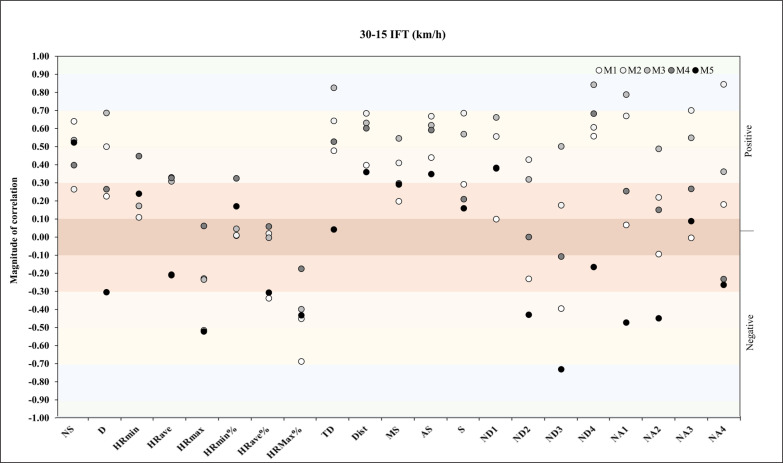
Correlation analysis between maximal aerobic speed and match physical and running demands.

Lastly, for the relationship between MAS and match physical and running demands, the analysis revealed a very large positive correlation with NA4 in M2 (*r* = -.84, *p* = .01). In M3, correlation analysis showed a very large positive correlation with TD, ND4, and NA1 (*r* = -.76, *p* = .03, *r* = -.71, *p* = .05, *r* = -.71, *p* = .05) (Figure 9).

**FIG. 10:**
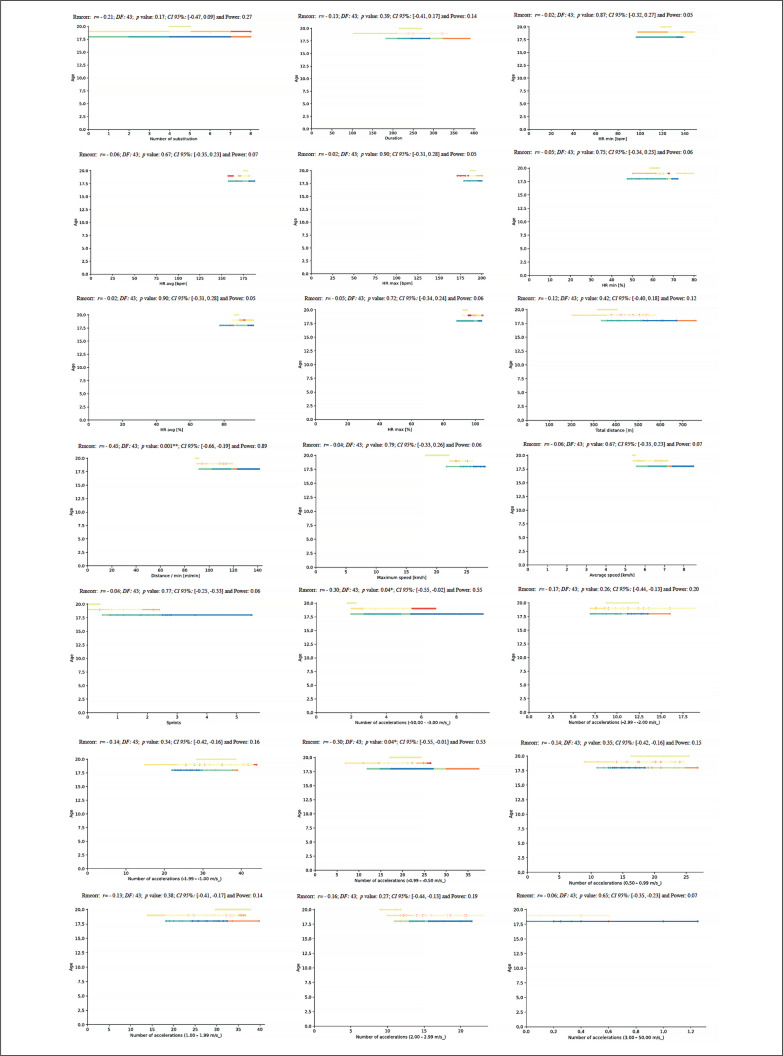
Repeated measures correlation analysis between age and match running demands during the congested schedule of futsal matches. Rmcorr for values (and corresponding *p*-values).

A repeated measures correlation was performed to evaluate the intra-individual association between anthropometric measures and physical fitness values and match running demands during the congested schedule of futsal matches considering within-subjects association and using the degrees of freedom (DF). An rmcorr repeated measures correlation analysis between age and match running demands during the congested schedule of futsal matches revealed a very large positive correlation for Dist (*rmcorr:* 0.45, *DF*: 43, *p* = .001, *CI 95%:* [-0.66, -0.19] and power: 0.89), and a moderate positive correlation for NA1 and NA4 (*rmcorr:* – 0.30, *DF:* 43, *p* = 0.04, *CI 95%:* [-0.55, -0.02] and power: 0.55 and *rmcorr:* – 0.30, *DF:* 43, *p*: 0.04; *CI 95%:* [-0.55, 0.01] and power: 0.53, respectively) (Figure 10).

**FIG. 11:**
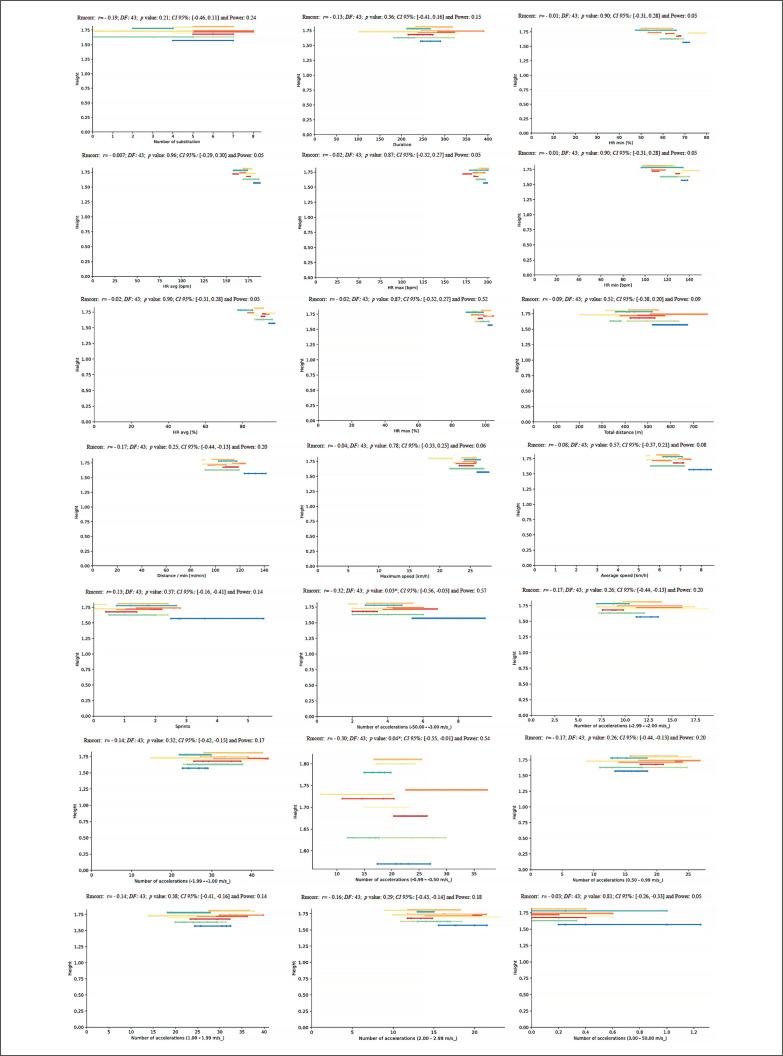
Repeated measures correlation analysis between height and match running demands during the congested schedule of futsal matches. Rmcorr for values (and corresponding *p*-values).

A new repeated measures correlation analysis was performed between height and match running demands during the congested schedule of futsal matches and only showed a moderate positive correlation for NA1 and NA4 (*rmcorr*: – 0.32, *DF:* 43, *p*: 0.03, *CI 95%:* [-0.56, -0.03] and power: 0.57, and *rmcorr*: -0.30, *DF:* 43, *p*: 0.04, *CI 95%:* [-0.55, -0.01] and power: 0.54, respectively) (Figure 11).

**FIG. 12:**
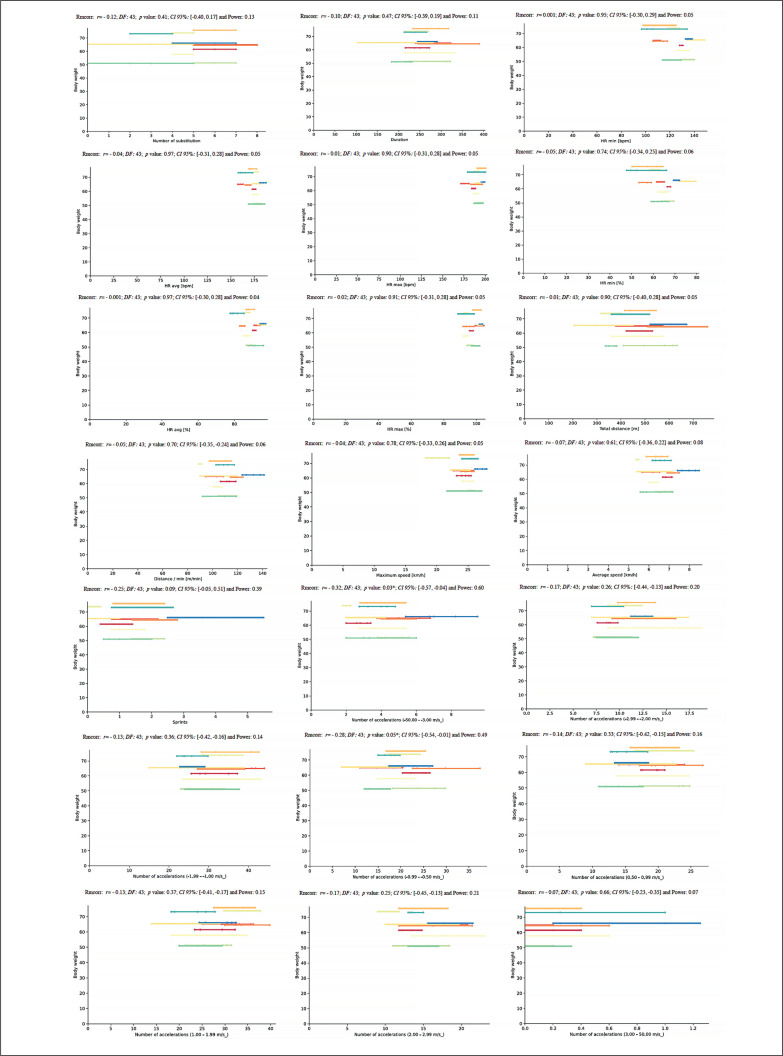
Repeated measures correlation analysis between body weight and match running demands during the congested schedule of futsal matches. Rmcorr for values (and corresponding *p*-values).

Another repeated measures correlation analysis was performed between body weight and match running demands during the congested schedule of futsal matches and revealed a moderate positive correlation for NA1 and NA4 (*rmcorr*: – 0.31; *DF:* 43, *p*: 0.03, *CI 95%:* [-0.56, -0.03] and power: 0.57, and *rmcorr*: – 0.30; *DF:* 43, *p*: 0.04, *CI 95%:* [-0.55, -0.01] and power: 0.53, respectively) (Figure 12).

**FIG. 13:**
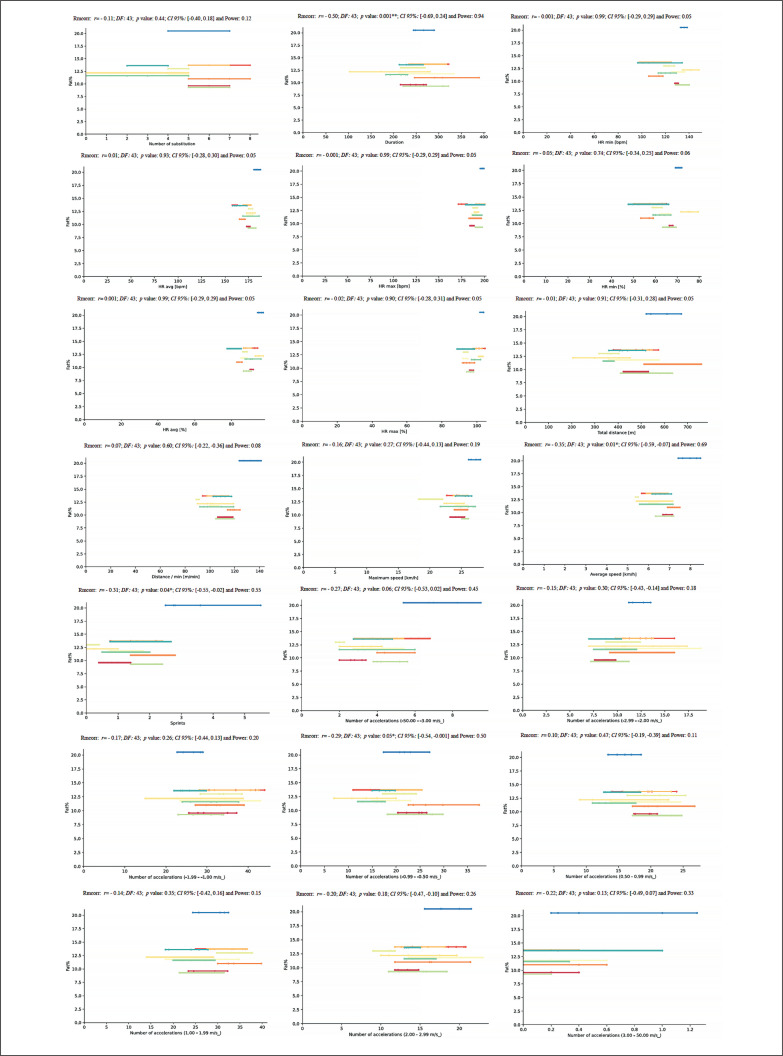
Repeated measures correlation analysis between fat% and match running demands during the congested schedule of futsal matches. Rmcorr for values (and corresponding *p*-values).

Along the same line, a further repeated measures correlation analysis was performed between fat% and match running demands during the congested schedule of futsal matches. It showed a moderate positive correlation for D, AS, S, and NA4 (*rmcorr*: – 0.50, *DF:* 43, *p*: 0.001, *CI 95%:* [-0.69, 0.24] and power: 0.94; *rmcorr*: – 0.35, *DF:* 43, *p*: 0.01, *CI 95%:* [-0.59, -0.07] and power: 0.69; *rmcorr*: – 0.31, *DF:* 43, *p*: 0.04, *CI 95%:* [-0.55, -0.02] and power: 0.55 and *rmcorr*: – 0.29, *DF:* 43, *p* value: 0.05, *CI 95%:* [-0.54, -0.001] and power: 0.50, respectively) (Figure 13).

**FIG. 14:**
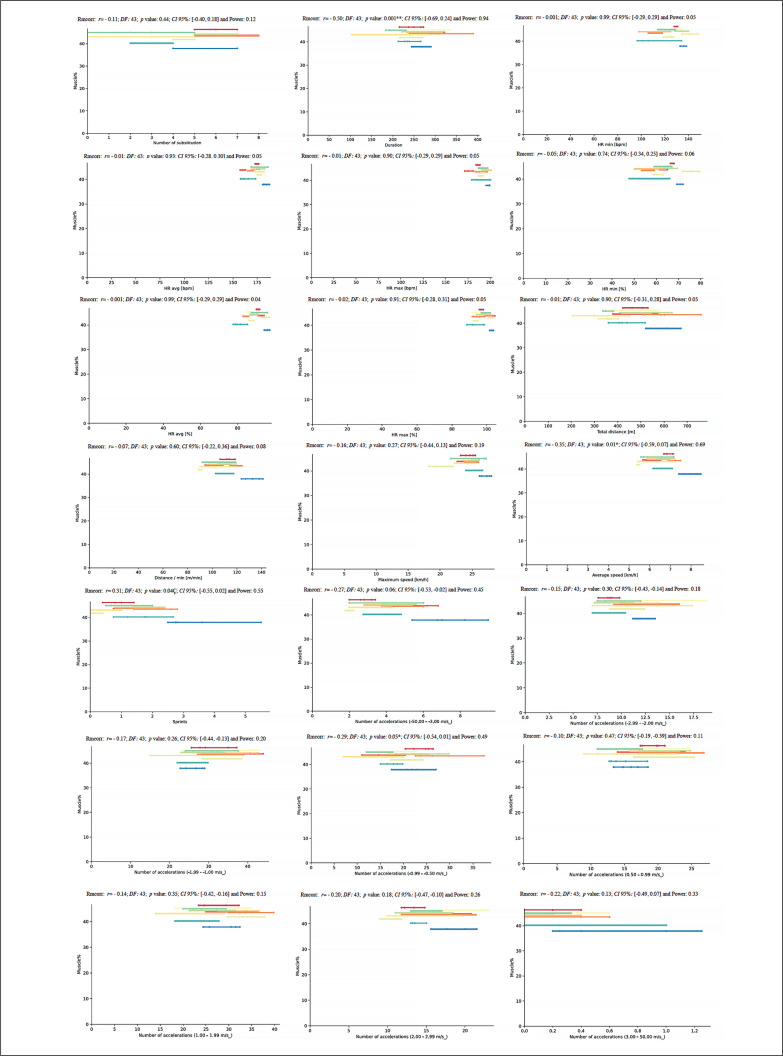
Repeated measures correlation analysis between muscle% and match running demands during the congested schedule of futsal matches. Rmcorr for values (and corresponding *p*-values).

Another repeated measures correlation analysis was performed between muscle% and match running demands during the congested schedule of futsal matches. It revealed a moderate positive correlation for NA1 and NA4 (*rmcorr*: – 0.32, *DF:* 43, *p*: 0.03, *CI 95%:* [-0.57, -0.04] and power: 0.60, and *rmcorr*: -0.28, *DF:* 43, *p*: 0.05, *CI 95%:* [-0.54, 0.01] and power: 0.49, respectively) (Figure 14).

**FIG. 15:**
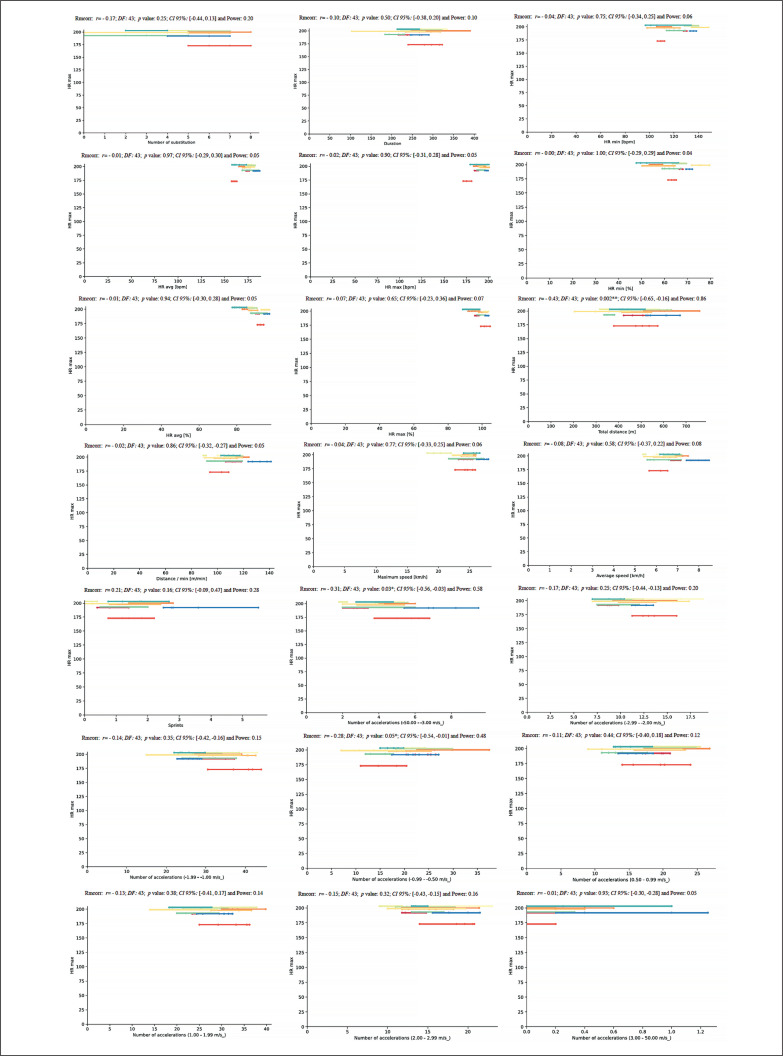
Repeated measures correlation analysis between HR_max_ and match running demands during the congested schedule of futsal matches. Rmcorr for values (and corresponding *p*-values).

A new repeated measures correlation analysis was performed between HR_max_ and match running demands during the congested schedule of futsal matches and revealed a very large positive correlation for TD (*rmcorr*: – 0.43, *DF:* 43, *p*: 0.002, *CI 95%:* [-0.65, -0.16] and power: 0.86) and a moderate positive correlation for NA1 and NA4 (*rmcorr*: – 0.31, *DF:* 43, *p*: 0.03, *CI 95%:* [-0.56, -0.03] and power: 0.58, and *rmcorr*: – 0.28, *DF:* 43, *p*: 0.05, *CI 95%:* [-0.54, 0.01] and power: 0.48, respectively) (Figure 15).

**FIG. 16:**
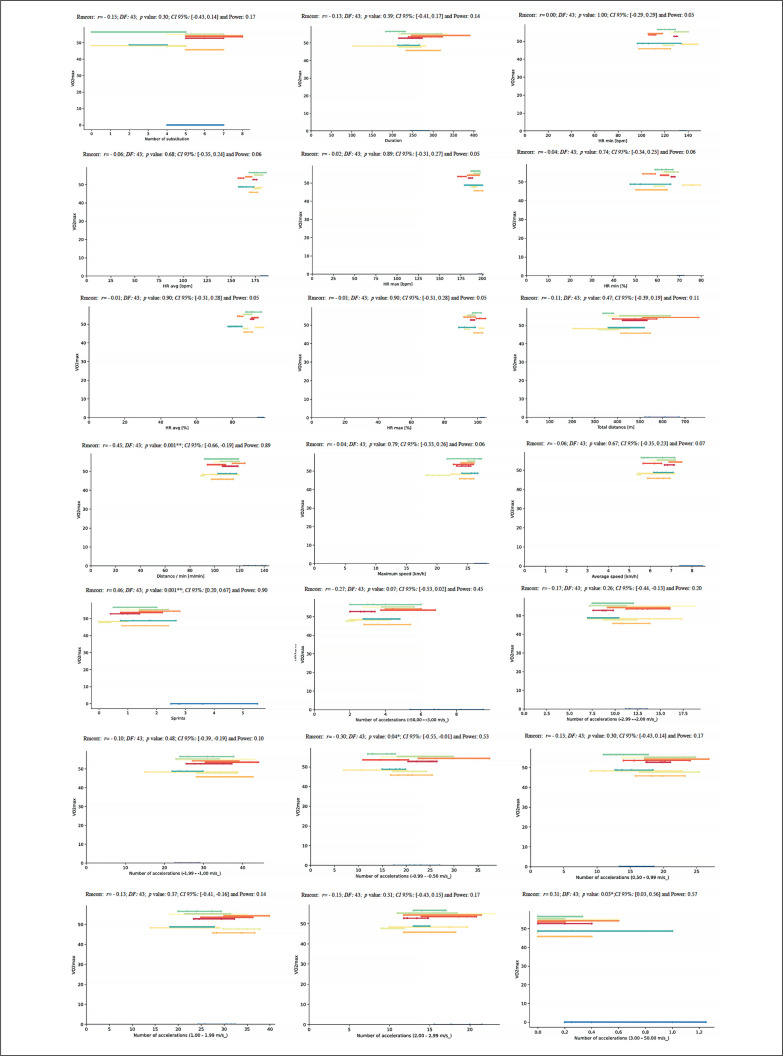
Repeated measures correlation analysis between V̇O_2max_ and match running demands during the congested schedule of futsal matches. Rmcorr for values (and corresponding *p*-values).

Lastly, for the relationship between V̇O_2max_ and match running demands, the repeated measures correlation analysis revealed an almost perfect correlation for S (*rmcorr*: 0.46, *DF:* 43, *p*: 0.001, *CI 95%:* [0.20, 0.67] and power: 0.90) and a moderate positive correlation for ND4 and NA4 (*rmcorr*: – 0.30, *DF:* 43, *p*: 0.04, *CI 95%:* [-0.55, -0.01] and power: 0.53 and *rmcorr*: 0.31, *DF:* 43, *p*: 0.03, *CI 95%:* [0.03, 0.56] and power: 0.57, respectively) (Figure 16).

## DISCUSSION

The present study aimed to analyse the match physical and running demands among matches and to examine the relationships between body composition, physical fitness measures, and match physical and running demands during a congested week of a futsal training camp. The main findings were that only the sprint, deceleration, and acceleration performance significantly differed among matches. Also, the overall body composition and physical fitness measures had large to very large correlations with match-running demands during congested periods of futsal competition.

Although there is no evidence regarding the differences between match physical and running demands of congested weeks in futsal, a recent systematic review reported that significant differences could be observed in the overall match running demands between match halves [43]. A decrease was observed from the first to the second half when considering the total volume of each external load measure. On the other hand, an increase was observed from the first to the second match half when considering the external load measures relativized per minute (e.g., according to each player’s time of match participation) [44]. Thus, the analysis of the match running demands should be relativized per minute, which was not the case in the present study and is considered as a limitation. However, in the present study, such differences were not present between matches during congested weeks, except for sprints. Our findings regarding the lack of differences among matches agree with a previous study that reported no significant differences for any external load measure [39]. However, that study [39] examined the differences between training sessions and not between matches, which may limit our inferences and extrapolation to the differences between matches.

Previous studies on different team sports have reported the influence that body composition has on physical fitness performance [45]. The literature focuses on body composition and anthropometric measures’ impact on physical performance [45]. However, a few studies have tested the associations between anthropometric measures and match-running demands in team sports. From the available evidence regarding the dependencies of match-running demands on body composition, it was reported that the sprinting distances covered were largely and negatively associated with the percentage of change of fat and lean mass [20, 21]. However, our findings contrast with those previously reported [20, 21]. Indeed, we did not observe any significant correlation between the fat% and sprint performance across the five matches. As a greater fat mass negatively affects sprint performance, it would be expected for positive correlations to be observed between them. A possible explanation may be related to the fact that the players maintained a relatively stable frequency of sprint volume during the five matches, as previously reported [46].

Regarding the relationships between physical fitness measures and match-running demands, our findings can be easily attributed to the interference of randomness, and it is. However, one can make a deterministic inference with a degree of uncertainty regarding our findings. First, a higher HR_max_ is one of the factors influencing a decrease in acceleration activities only during the last match of a congested week. This finding may be attributed, among others factors, to acute (e.g., short-term) mental fatigue, which causes an increase in sympathetic activity and decreases parasympathetic nerve activity [47]. This is particularly interesting for coaches to consider when selecting players with high match participation in congested periods. An athlete with a lower HR_max_ is more susceptible to poor heart rate responses when exposed to high-intensity stimuli during a specific period [48]. As a complement to the above statement, a greater level of V̇O_2max_ is positively associated with greater acceleration activities and TD in M2 and M3.

Our findings suggest that a greater 30–15 IFT performance is associated with greater activity of high-intensity accelerations during M2. On the other hand, a greater performance on the 30–15 IFT is associated with decreased activity of low-intensity accelerations and TD during M3. A previous study [39] conducted on sixteen professional football players revealed large to very large correlations between the 30–15 IFT performance and TD (*r* = 0.69 to 0.87), high-intensity running (*r* = 0.66 to 0.75), and accelerations and decelerations (*r* = 0.56 to 0.68), which is somewhat consistent with our findings. However, that study was conducted on football players and not on futsal. Also, the study methodology had a very different approach to our study, as the authors examined such associations using small-sided games [39]. In the case of the futsal context, and to the best of the authors’ knowledge, only one study tested the relationships mentioned above in futsal players [18]. That study showed that the 30 m sprint test could be explained by HR_ave_, HR_max_, dist/min, MS, and AS between 38 and 48%.

Moreover, a recent study investigated the interplay between weekly training load and performance outcomes in a professional male futsal team over two seasons [49]. The study analysed training load, recovery status, well-being, neuromuscular performance variability, and match performance. Notably, the study’s path analysis model elucidated that this framework accounted for 31% of team performance, highlighting the intricate connections between training load, neuromuscular performance, and match outcomes. The findings underscore the importance of monitoring variables such as neuromuscular performance variability in comprehending player and team performance dynamics.

The present study presents some limitations that need to be addressed. One of the main limitations is related to the small sample used, which does not allow the generalization of the results. The fact that the physical fitness assessments were carried out only once (at the beginning of the training camp) limits the power of the correlations. The concise time frame of the observations and the fact that it was conducted during a training camp were also among the main limitations of the present study. Future studies should consider observing an entire futsal season to improve the power of the present findings.

## CONCLUSIONS

During a futsal training camp of eleven days with five futsal matches, no significant differences in match running demand measures were found, excepted for sprint performance. The baseline body composition and the overall physical fitness measures may be essential factors in matching running demands during a congested futsal match period. Futsal players who perform better in the 30–15 IFT can sustain a greater volume of high-intensity accelerations during a congested period.
